# Time-dependent variation in immunoparalysis biomarkers among patients with sepsis and critical illness

**DOI:** 10.3389/fimmu.2024.1498974

**Published:** 2024-12-06

**Authors:** Abigail Samuelsen, Erik Lehman, Parker Burrows, Anthony S. Bonavia

**Affiliations:** ^1^ Department of Anesthesiology and Perioperative Medicine, Penn State Medical Center, Hershey, PA, United States; ^2^ Department of Public Health Sciences, Penn State College of Medicine, Hershey, PA, United States; ^3^ Critical Illness and Sepsis Research Center, Penn State College of Medicine, Hershey, PA, United States

**Keywords:** sepsis, critical illness, immune monitoring, immunodeficiency, human leukocyte antigen-DR, lymphocyte count, immunoparalysis, HLA-DR

## Abstract

**Introduction:**

Immunoparalysis is a state of immune dysfunction characterized by a marked reduction in the immune system’s responsiveness, often observed following severe infections, trauma, or critical illness. This study aimed to perform a longitudinal assessment of immune function over the initial two weeks following the onset of sepsis and critical illness.

**Methods:**

We compared ex vivo-stimulated cytokine release from whole blood of critically ill patients to traditional markers of immunoparalysis, including monocyte Human Leukocyte Antigen (mHLA)-DR expression and absolute lymphocyte count (ALC). A total of 64 critically ill patients were recruited in a tertiary care academic medical setting, including 31 septic and 33 non-septic patients.

**Results:**

While mHLA-DR expression significantly increased over time, this was primarily driven by the non-septic subset of critically ill patients. ALC recovery was more pronounced in septic patients. Ex vivo stimulation of blood from septic patients revealed significant increases in TNF and IL-6 production over time. However, interferon-gamma production varied depending on the ex vivo stimulant used, and after normalization of cytokine concentrations to lymphocyte counts, it did not show significant recovery over time from illness onset. No significant correlation was found between mHLA-DR expression and other immunoparalysis biomarkers.

**Discussion:**

These findings suggest the need for more nuanced immune monitoring approaches beyond the traditional ‘sepsis’ versus ‘non-sepsis’ classifications in critically ill patients. Additionally, they provide further evidence of a potential window for targeted immunotherapy in the first weeks of critical illness.

## 1 Introduction

Sepsis, marked by host immune dysregulation and subsequent organ dysfunction, is a leading cause of critical illness, often resulting in death within days to months after the onset of acute illness (1). Patient mortality rates in the intensive care unit (ICU) have been reported to be as high as 30% (2–5). Furthermore, secondary infection, resulting from sepsis-induced impairment of host immunity, is a well-recognized cause of morbidity (6). One study found 69% of hospital readmissions were related to infection, with over 50% of those as recurrent or unresolved infections (7). This ‘immunoparalysis’ is multifactorial (8, 9), driven by immune cell exhaustion and apoptosis (10–12), anti-inflammatory cytokine production (13–15), metabolic dysfunction (16, 17), and the expansion of regulatory T cells (18–21) and myeloid-derived suppressor cells (22, 23).

Significant efforts have been made to identify immune phenotypes in sepsis to develop clinically useful risk-stratification tools that could improve clinical outcomes. A recent landmark study by Venet and the REALISM (REAnimation Low Immune Status Marker) investigators identified a subgroup of severely injured patients who developed delayed injury-acquired immunodeficiency, independent of the primary disease (24). These findings suggest that immunoparalysis is not specific to sepsis but can be assessed in critically ill patients by monitoring a common panel of pro-/anti-inflammatory markers indicative of innate/adaptive immune responses. Moreover, this research highlights the urgent need to identify routinely accessible immunosurveillance markers to pinpoint patients who might benefit from customized immunoadjuvant therapies (25). Given the rapid progression of sepsis and the narrow window for effective clinical intervention, it is also important that these biomarkers can be rapidly processed in a point-of-care clinical setting.

Based on these observations, our laboratory has been developing a rapid and precise assay for detecting immunoparalysis. The clinical implications would be that early identification of these high-risk patients may allow therapeutic intervention that would boost patients’ immune response and avoid disease-associated morbidity. In a recent study, we demonstrated that ex vivo stimulation of both innate and adaptive immune components did not predict secondary infection rates. However, it reliably predicted organ dysfunction developing within 48 h of the assay (26). Clustering analysis also revealed two distinct immune phenotypes, characterized by differential responses to 18 h of lipopolysaccharide (LPS, also known as endotoxin) stimulation and 4 h of anti-CD3/anti-CD28 stimulation (26). Despite these advancements, we have not yet conducted a direct comparison between this ex vivo immune-phenotyping method and traditional markers of immunoparalysis, such as monocyte Human Leukocyte Antigen-DR (mHLA-DR) expression and absolute lymphocyte count (ALC) (27).

mHLA-DR and ALC are key biomarkers for evaluating immune dysfunction in sepsis, reflecting both innate and adaptive immune suppression, respectively. Reduced mHLA-DR expression indicates monocyte deactivation, increasing the risk of secondary infections, with studies showing its predictive value for mortality in septic patients (28). Venet and Monneret (29) further suggest that mHLA-DR can stratify patients who may benefit from immunostimulatory therapies. ALC, a marker of adaptive immunity, reflects lymphopenia from T-cell and B-cell apoptosis, which has been linked to higher mortality in sepsis (30) and poor outcomes overall (31). Together, mHLA-DR and ALC provide a more comprehensive view of immunoparalysis in sepsis, enhancing prognostic accuracy and possibly aiding in the identification of patients suitable for targeted immunotherapy (29, 31).

The primary goals of this analysis were to perform a comparative evaluation of these biomarkers and to address the gap in understanding immune trajectories during the transition from acute to subacute phases of critical illness. Specifically, we aimed to compare ex vivo cytokine release from whole blood samples of critically ill patients to ALC and mHLA-DR expression over the two weeks following the onset of acute illness. We hypothesized that patterns of ex vivo-stimulated cytokine release would mirror the trajectories of mHLA-DR expression and ALC over time. Furthermore, we hypothesized that patients with immunoparalysis would have poorer clinical outcomes. Given the inherent challenges in reliably diagnosing secondary infections, including inconsistent diagnostic criteria and underreporting (30, 32, 33), we defined immunoparalysis solely based on immune parameters rather than clinical diagnosis.

## 2 Results

### 2.1 Study population

We recruited a total of 64 critically ill patients, including 31 individuals diagnosed with sepsis and 33 non-septic control (CINS) patients. A detailed breakdown of the demographic characteristics and clinical outcomes for these patients is provided in **Table 1**. For septic patients with confirmed infections, the microbial sources of infection are listed in **Supplementary Table S1**. The characteristics of the CINS control group are comprehensively detailed in **Supplementary Table S2**. During the 30-day follow-up period, only two septic patients and two CINS patients were lost to follow-up (**Supplementary Figure S1**).

**Table 1:** Patient demographics and clinical outcomes.

Demographic and Clinical Characteristics	Critically Ill Septic (n = 31)	Critically Ill Non-Septic (n = 33)	P-value
Age (mean ± SD)	65.5 ± 13.4	64.1 ± 15.4	0.708
Female (n, %)	14 (48)	15 (52)	0.981
Infection etiology (n %)			
—Gram-negative	11	N/A	
—Gram-positive	9	N/A	
—Mixed	11	N/A	
—Clinical diagnosis only, no positive cultures	2	N/A	
Severity of illness			
APACHE II Score (mean ± SD)	21.1 ± 7.1	18.4 ± 6.7	0.123
SOFA Score (median, IQR)	8.0 (7.1 – 9.7)	6.0 (5.7 – 7.9)	0.042
Charlson Comorbidity Index (median, IQR)	5.0 (4.0 – 5.9)	4.0 (3.0 – 4.9)	0.113
Patients receiving stress-dosed hydrocortisone ≥ 8 h (n, %)	9 (29.0)	5 (15.2)	0.179
Daily hydrocortisone dose (mg) (median, IQR)	150 (119 – 188)	200 (129 – 264)	0.202
Duration of hydrocortisone (days*) (median, IQR)	3.0 (1.9 – 3.9)	2.0 (0.6 – 3.8)	0.374
Laboratory Values			
Leukocyte Count x10^3^/µl (median, IQR)	14.5 (13.4 – 20.7)	10.8 (10.7 – 14.8)	0.055
Absolute Lymphocyte Count x10^3^/µl (median, IQR)	0.66 (0.54 – 0.78)	1.01 (0.87 – 1.54)	<0.001
Absolute Monocyte Count x10^3^/µl (median, IQR)	0.47 (0.34 – 0.68)	0.78 (0.74 – 1.15)	<0.001
Lactic Acid (mg/dL) on admission (median, IQR)	3.8 (3.2 – 4.9)	2.7 (2.2 – 4.2)	0.112
Shock (vasopressors and lactate ≥ 2) on admission (n %)	10 (32.3)	10 (30.3)	0.866
Short-Term Outcomes			
Proportion of Cohort developing Secondary Infections (n %)	1 (3.2)	2 (6.1)	1.000
30-Day Mortality Rate (n, %)	3 (4.7)	6 (9.4)	0.478
Hospital Length of Stay days (median, IQR)	10.0 (9.5 – 17.4)	8.0 (8.3 – 21.0)	0.472
30-Day Hospital readmission among survivors (alive/30-day survivors, %)	4/28 (14.3)	2/25 (8.0)	0.423
Discharged status			
—Home (n, %)	13 (22.0)	15 (25.4)	0.880
—Skilled nursing facility (n, %)	13 (22.0)	11 (18.6)	0.393
—Long-term acute care hospital (n, %)	2 (3.4)	1 (1.7)	0.592
—Hospice (n, %)	0	1 (1.7)	1.000

*within 14 days following enrollment. P-values represent two-sample t-test comparing group means for normally distributed variables, Wilcoxon Rank Sum test comparing group medians for skewed variables, or Chi-square test comparing proportions for categorical variables.

N/A, Not Applicable.

### 2.2 Rates of mHLA-DR recovery do not differ between critically ill patients with and without sepsis

**Figure 1:**
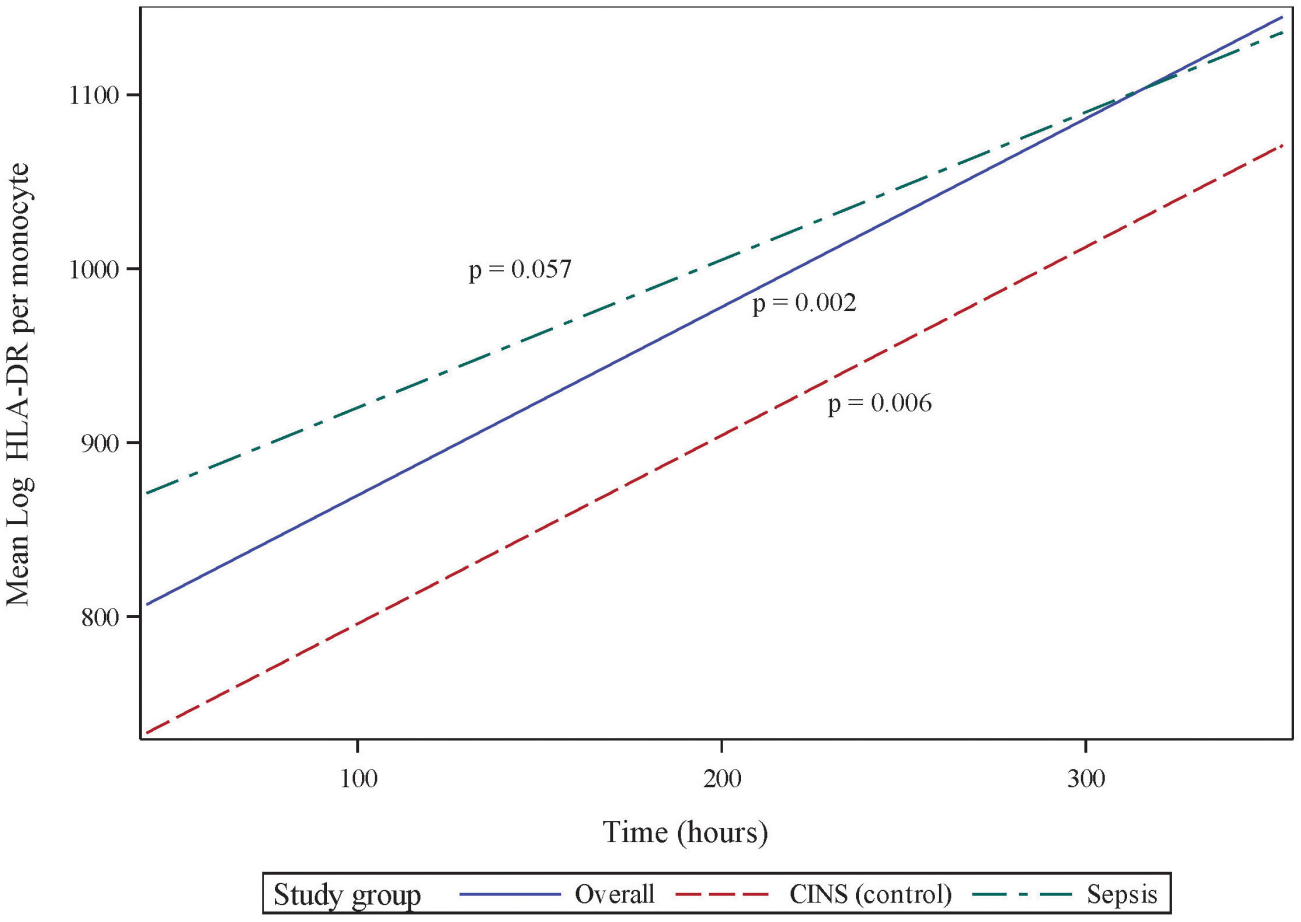
Monocyte Human Leukocyte Antigen (mHLA)-DR expression over the initial 336 h (14 d) following critical illness. *P-*values indicate the significance of the rate of change in mHLA-DR expression in each subgroup over time, as analyzed using a mixed-effects linear regression model.

While mHLA-DR expression significantly increases over the first 336 h (14 d) following onset of critical illness (p = 0.002), this increase is driven primarily by immune responses in non-septic patients (p = 0.006), with septic patients exhibiting only a trend toward significance over this time (p = 0.057, **Figure 1**). There was no significant difference between the slopes (i.e., rates of immune recovery, as assessed by mHLA-DR expression over the 14-day period) of critically ill patients with or without sepsis (p = 0.688).

### 2.3 Rates of absolute lymphocyte count recovery do not differ between critically ill patients with and without sepsis

**Figure 2:**
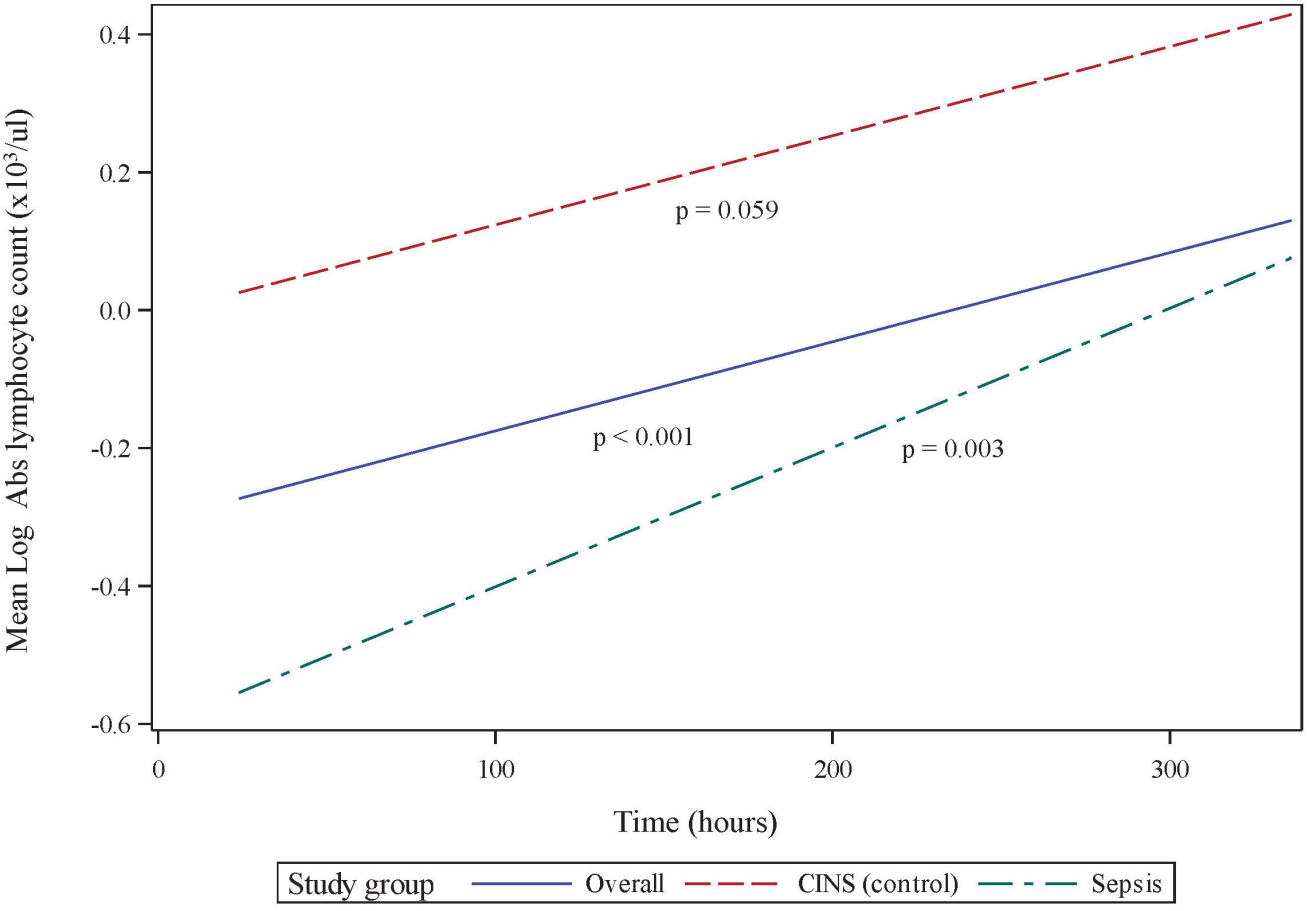
Absolute lymphocyte count (ALC) over initial 336 h (14 d) following critical illness. *P-*values indicate the significance of the rate of change in ALC in each subgroup over time, as analyzed using a mixed-effects linear regression model.

Absolute lymphocyte count (ALC) also significantly recovers over the 336 h (14 d) following onset of critical illness (p <0.001, 95% CI 0.072 - 0.259, **Figure 2**). However, in contrast to the pattern of mHLA-DR expression, this recovery is primarily driven by the septic subset of critically ill patients (p = 0.003, 95% CI 0.073 - 0.332), with the CINS subgroup exhibiting a trend toward significance over this period (p = 0.059). Although the rates of ALC recovery do not vary significantly between subgroups (p = 0.436), there are notable differences between the mean ALC values of each subgroup at 24 h and 168 h (7 d) (p <0.001 and p = 0.006, respectively). By 336 h (14 d), these differences are no longer significant (p = 0.195).

### 2.4 Recovery in tumor necrosis factor production, following ex vivo endotoxin stimulation of whole blood, occurs more rapidly following acute sepsis as compared with non-sepsis critical illness

**Figure 3:**
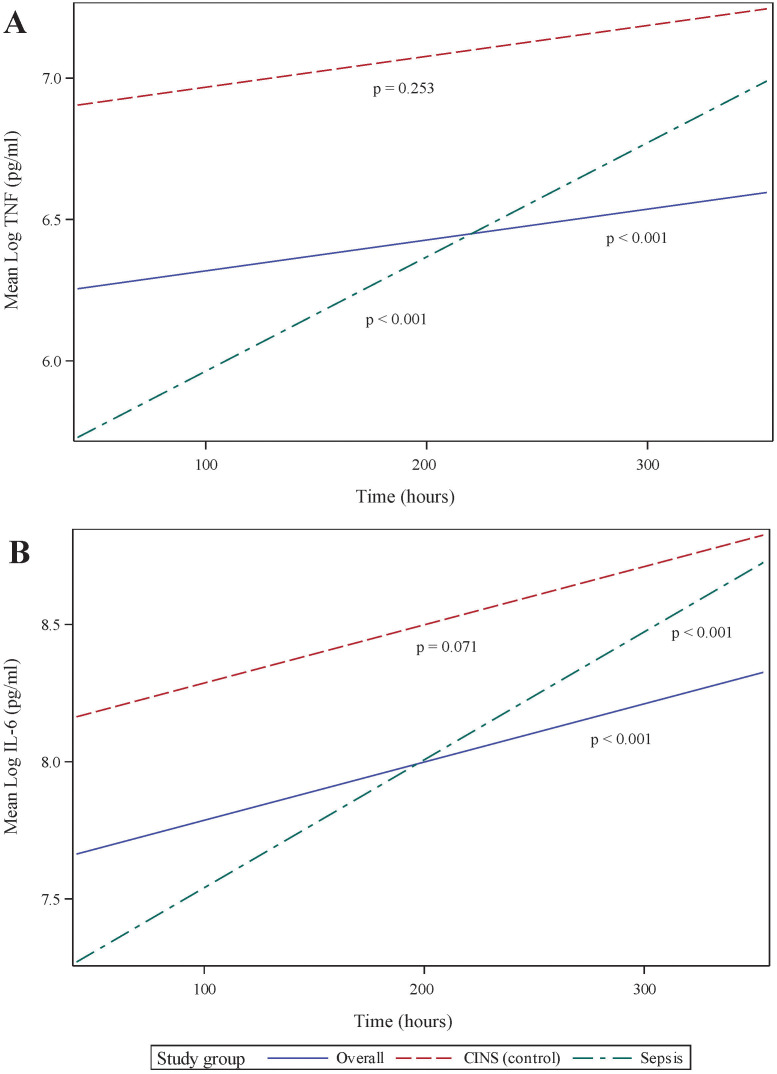
Ex vivo production of cytokines over time, following 18 h of endotoxin stimulation of whole blood sampled from critically ill patients. **(A)** Stimulated tumor necrosis factor (TNF) production, **(B)** Stimulated interleukin (IL)-6 production. *P-*values indicate the significance of the rate of change in cytokine concentrations in each subgroup over time, as analyzed using a mixed-effects linear regression model.

LPS-stimulated cytokine production recapitulates the pattern of immune recovery modeled by mHLA-DR and ALC (**Figure 3A**). Specifically, following 18 h of endotoxin stimulation, TNF production was observed to recover significantly over time in the combined cohort (p <0.001, 95% CI 0.115 - 0.398). TNF production in septic patients increased significantly over the 14-day observation period (p <0.001, 95% CI 0.195-0.615), although not in the CINS subset (p = 0.253). Importantly, TNF recovery occurred more rapidly in sepsis patients (p = 0.041), driven primarily by differences in the concentration of this cytokine at 24 h (p <0.001) and 168 h (p = 0.001) of critical illness.

The pattern of LPS-stimulated interleukin (IL)-6 was closely related to TNF production, although recovery rates did not differ between subgroups. In all critically ill patients taken together, IL-6 significantly increased over time (p <0.001, 95% CI 0.166 – 0.513, **Figure 3B**), primarily driven by immune responses in the septic subgroup (p <0.001, 95% CI 0.209 – 0.726). The CINS cohort demonstrated a trend toward significant IL-6 recovery over time (p = 0.071). Mean IL-6 production was significantly different between subgroups at the 24 and 168 h time points (p = 0.002 and 0.038, respectively), although the overall rate of IL-6 recovery was not significantly different between groups (p = 0.147).

### 2.5 Interferon-gamma response, following ex vivo stimulation of whole blood, varies by stimulant used

**Figure 4:**
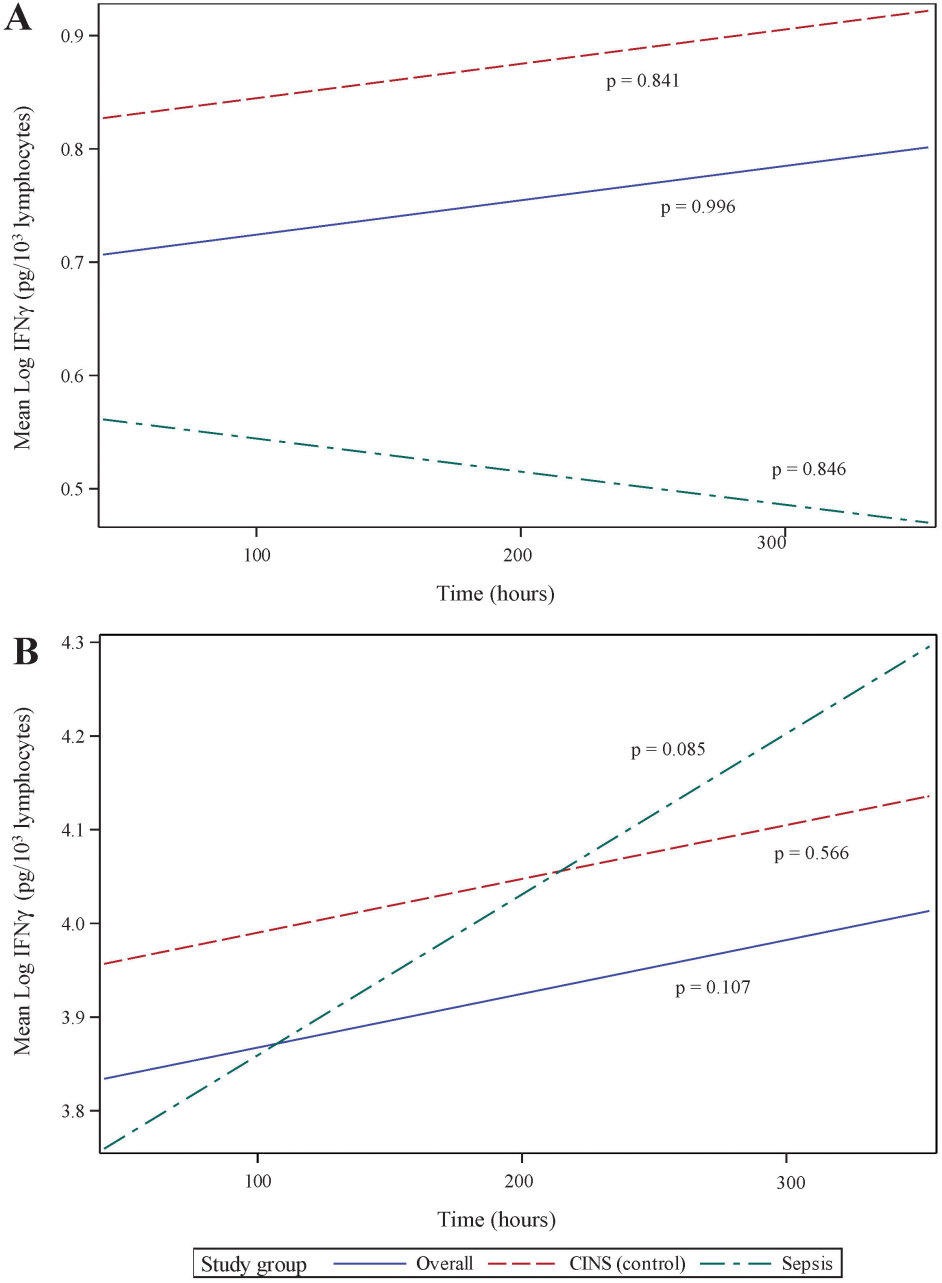
Ex vivo production of interferon (IFN)g over time, following 18 h of **(A)** anti-CD3/anti-CD28 antibody, and **(B)** PMA. IFNg concentrations have been normalized to lymphocyte count. *P-*values indicate the significance of the rate of change in IFNg concentrations in each subgroup over time, as analyzed using a mixed-effects linear regression model.

T lymphocyte responses play a crucial role in the adaptive immune response to sepsis. To assess these responses, we stimulated whole blood using anti-CD3 and anti-CD28 antibodies for 18 h. While this stimulation did not result in a significant overall difference in interferon (IFN)g production from 24 168 h following the onset of critical illness, we did observe specific differences between mean IFNg concentrations in each group **Figure 4A**. These differences were limited to 24 h (p = 0.048) and 336 h (p = 0.043) of critical illness.

Non-specific T lymphocyte stimulation, using PMA, demonstrated a more dramatic change in IFNg production over time. Specifically, cytokine production increased substantially over time in all patients (p <0.001, 95% CI 0.116 – 0.407) and in the septic subset (p = 0.003, 95% CI 0.12 – 0.552). The CINS subset demonstrated a less dramatic change in PMA-induced IFNg production (p = 0.059, 95% CI -0.008–0.383). As with anti-CD3/anti-CD28 stimulation, mean IFNg concentrations were most significant at 24 h (p = 0.006) and 168 h (p = 0.030) following critical illness onset.

Since, lymphopenia is a critical component of sepsis and critical illness (30, 34), we normalized IFNg production to cell count, thus distinguishing between quantitative and qualitative lymphocyte defects. When normalized to ALC, there was no statistically significant increase in stimulated IFNg production over time. This held true whether T lymphocytes were stimulated using specific (anti-CD3/anti-CD28, **Figure 4A**) or non-specific (PMA, **Figure 4B**) agents. It also implies that, during critical illness, there is quantitative recovery of lymphocyte counts (**Figure 2**) although no qualitative recovery.

### 2.6 mHLA-DR was not correlated with other measured markers of immunoparalysis

Using repeated measures correlation (r_rm_), we examined the relationships among various markers of immunoparalysis. These included: (1) mHLA-DR expression, (2) ALC, (3) stimulated cytokine production, and (4) stimulated cytokine production normalized to lymphocyte count (in the case of IFNg) and monocyte count (in the case of TNF).

Monocyte HLA-DR expression was not significantly associated with other immunoparalysis biomarkers at each time point. ALC demonstrated a weak positive correlation with endotoxin-stimulated TNF (r_rm_ = 0.336) and IL-6 (r_rm_ = 0.351) concentrations at each time point. Additionally, ALC was weakly, positively correlated with IFNg concentration following anti-CD3/anti-CD28 (r_rm_ = 0.385) and PMA (r_rm_ = 0.347) stimulation.

There was a moderate, positive correlation between endotoxin-stimulated cytokines TNF and IL-6 w(r_rm_ = 0.495), with a higher correlation observed within the non-septic subset (r_rm_ = 0.576). IFNg concentration following T cell-specific (anti-CD3/anti-CD28) and non-specific (PMA) stimulation was also positively correlated (r_rm_ = 0.446 in all patients, and 0.523 in CINS subset). When normalized to lymphocyte count, these responses to T cell stimulants remained moderately correlated (r_rm_ = 0.359 in all patients, and 0.529 in CINS subset).

### 2.7 While biomarkers of immunoparalysis are not associated with secondary infection rate, endotoxin-associated IL-6 production is associated with 30-day hospital readmission

No association was found between biomarker concentrations and either 30-day mortality or the number of days from the onset of critical illness until death. Additionally, there was no observed association with 30-day secondary infection rates. In the analysis of hospital length of stay following critical illness with or without sepsis diagnosis, we found statistically significant intercepts in several quantile regression models, though these were not accompanied by associations with any of the independent variables. These findings may reflect an intrinsic difference in hospital length of stay between sepsis and CINS patients, not explained by the biomarkers used in this investigation.

Logistic regression revealed a statistically significant relationship between endotoxin-induced IL-6 concentration and 30-day hospital readmission (p = 0.015). Specifically, for every 100-fold increase in IL-6 concentration there was a 4.3% increase in the odds of 30-day hospital readmission. Additionally, there was a significant association between the sepsis versus CINS subgroups and hospital readmission (p = 0.045), with sepsis patients showing markedly higher odds of 30-day readmission compared to CINS controls (odds ratio = 27.616). While the total number of hospital readmissions was low (four readmissions among 28 septic patients who were alive at 30 days, and 2 readmissions among 25 CINS patients who were alive at 30 days), these findings may suggest that elevated endotoxin-induced IL-6 concentrations and sepsis status are important predictors of readmission.

## 3 Discussion

Venet et al. recently reported significant immune changes by the end of the first week post-hospital admission for severe injury. These changes were associated with an increased risk of secondary infections and were termed ‘delayed injury-acquired immunodeficiency’ (24). In a similar, though smaller, prospective observational analysis, we delved deeper into different immune surveillance markers expressed during the first two weeks of critical injury. The concentrations of all immunoparalysis biomarkers employed in the present study increased over this recovery period. When comparing sepsis and CINS subgroups, however, we noted that temporal increases in mHLA-DR were primarily driven by the CINS subset of patients, while the septic subset drove increases in ALC, LPS-induced TNF and IL-6 (whether normalized to monocyte count or not), and PMA-induced IFNg in the overall cohort.

In a recent, retrospective analysis, Adigbli et el. reported that early, persistent lymphopenia (defined as ALC <1.0 × 10^9^/L on at least 2 days within the first 4 days of ICU admission) was associated with increased risk of death in critically ill patients with and without sepsis, with the former group demonstrating a stronger association (hazard ratios 1.89 and 1.17, respectively) (35). Using data from patients who had ALC measured on days 1 and 7 of critical illness, we did not observe a similar association. This difference may be attributed to our smaller cohort size. Mean ALC was significantly different between sepsis and CINS subgroups within the first week following enrollment, although there was no difference between groups at day 14. Surprisingly, despite lymphocytes being the predominant cellular source of IFNg in blood, ALC was only weakly correlated with stimulated IFNg production over time. However, patterns of IFNg produced in response to anti-CD3/anti-CD28 and PMA remained moderately correlated, indicating primarily T cell production.

The pattern of cytokines produced in response to ex vivo stimulation of whole blood with endotoxin mirrored the changes observed in the ALC. Specifically, mean TNF concentrations significantly differed between sepsis and CINS subgroups on days 1 (p <0.001) and 7 (p = 0.001), but not on day 14. Similarly, mean IL-6 concentrations showed significant differences between subgroups on days 1 (p = 0.0034) and 7 (p <0.001), with no significant difference on day 14 (p = 0.147). Specific T lymphocyte stimulation resulted in a similar pattern in IFNg production (p = 0.048 on day 1 and p = 0.043 on day 7, with no significant difference on day 14). Non-specific T lymphocyte stimulation with PMA also reflected this pattern (p = 0.006 on day 1, p = 0.030 on day 7, and p = 0.493 on day 14). These findings suggest that the inflammatory response is acute and pronounced during the early stages of critical illness but tends to normalize by the second week following injury. Comparison of these biomarkers between healthy volunteers (or hospitalized and non-critically ill patients) and critically ill patients may shed further light on any residual deficits in immune function over this 14-day period.

Several prior studies have described mHLA-DR as a marker of monocyte deactivation or evolving immunoparalysis, especially in the context of sepsis (36, 37). Deactivated monocytes are characterized by a loss of antigen-presenting capacity and decreased reduction of their ability to produce endotoxin-induced TNF *in vitro* (25, 38). De Roquetaillade et al. found that a decrease or continued low expression of mHLA-DR within the first week of ICU admission was independently linked to a higher risk of subsequent infections (39). However, mHLA-DR has several critical shortcomings that preclude its use as a biomarker in the clinical setting, including its dependence on flow cytometry and user variability in measurement and analysis (40, 41). In this respect, ALC or ex vivo assays using rapid and automated cytokine measurement platforms may offer a feasible alternative for point-of-care-testing.

In this study, we observed significant recovery of mHLA-DR expression over time, particularly in the CINS subgroup. However, considering the findings by Venet et al. (24), a shift beyond the traditional ‘sepsis’ versus ‘non-sepsis’ classifications may provide a more nuanced approach to understanding immunoparalysis in critically ill patients. This rationale guided our decision not to investigate the bacterial-specific marker, procalcitonin, in the present investigation. While useful for identifying infection, procalcitonin may not comprehensively capture the broader immune suppression dynamics associated with immunoparalysis in critically ill patients (42–45).

This study has several important limitations that must be acknowledged. First, 15% of patients in the CINS group and 29% in the sepsis group received at least one dose of hydrocortisone more than 8 hours prior to the ex vivo assay. While these proportions are notably lower than previously reported rates of steroid use in septic shock (46), it is crucial to recognize that the use of corticosteroids may have influenced the observed immune responses. Given that this is an observational study conducted in a tertiary care medical center, we believe that including these patients in our analysis provides a realistic representation of critically ill patients who could potentially benefit from immune-adjuvant therapies aimed at improving clinical outcomes. This inclusion mirrors the diverse steroid use patterns seen in real-world settings and thus enhances the generalizability of our findings.

Second, the study design did not allow for the assessment of the functional capacity of immune cells beyond cytokine production. Further functional assays could provide a more comprehensive understanding of the immune status of critically ill patients. Third, our definition of immunoparalysis was based solely on immune parameters without considering clinical outcomes such as secondary infections. Although this approach standardizes the assessment of immunoparalysis, it may not fully capture the clinical relevance of the observed immune alterations. Despite these limitations, our findings provide valuable insights into the immune dynamics of critically ill patients with and without sepsis and highlight the need for more nuanced immune monitoring approaches.

## 4 Conclusions

This study demonstrates that immune function in critically ill patients varies significantly over the first two weeks following the onset of sepsis. Monocyte HLA-DR expression increased over time primarily in non-septic patients, while ALC and ex vivo cytokine production demonstrated temporal changes in septic patients. Furthermore, the rate of IFNg production after anti-CD3/anti-CD28 stimulation did not significantly differ between sepsis and non-sepsis groups over the study period, although the mean cytokine concentrations did show notable differences between groups.

These biomarker changes should not be interpreted as indicative of clinical recovery, as the relationship between these immune parameters and actual patient outcomes remains unclear. However, they highlight the complexity of immune trajectories in critical illness and underscore the need for further research to determine whether these biomarker trends have prognostic or therapeutic significance in septic and critically ill patients.

## 5 Materials and methods

### 5.1 Patient cohort

Critically ill patients potentially suffering from sepsis were identified using a Modified Early Warning Scoring (MEWS) algorithm from November 2021 to March 2024. To ensure an unbiased selection process, dual, independent investigators reviewed electronically flagged patient records to identify those meeting inclusion criteria. Informed consent was obtained from patients with decision-making capacity or from legally authorized healthcare representatives for those without decision-making capacity.

Eligible participants were adults over the age of 18, recruited within 48 h of the onset of critical illness. Sepsis was defined according to the Sepsis-3 criteria (1). Critical illness was defined by the need for ongoing noninvasive or invasive respiratory support and/or continuous intravenous vasopressor medications. Non-survivors were those who died within 30 days of enrollment. Patients receiving immunomodulating therapies were excluded. Measured clinical outcomes included 30-day mortality, in-hospital mortality, secondary infection rates, ICU length-of-stay and hospital readmission rates.

Arterial or venous blood was collected in sodium-heparin tubes at day 1 (time of enrollment), and subsequently at days 7 and 14 in survivors.

### 5.2 Cytokine responses by whole blood following ex vivo stimulation

Fifty microliters of whole blood were diluted ten-fold in HEPES-buffered Roswell Park Memorial Institute (RPMI) 1640 media and exposed to specific stimulants as per established protocols (14, 15). Blood samples from each participant were treated under three conditions: (1) 500 pg/mL lipopolysaccharide (LPS) from Salmonella enterica strain abortus equi, (2) 500 ng/mL anti-CD3 with 2.5 µg/mL anti-CD28, or (3) 10 ng/mL phorbol 12-myristate 13-acetate (PMA) with 1 µ/mL ionomycin. After incubation for 18 h at 37°C with 5% CO2, concentrations of IFNg, TNF, and IL-6 were measured in triplicate using the Ella™ automated immunoassay system (Bio-Techne, Minneapolis, MN). Data were processed using Simple Plex Runner software v.3.7.2.0 (Bio-techne) and were available within 90 minutes.

The production of TNF was normalized to the monocyte count, and IFNg production was normalized to the lymphocyte count, based on daily complete blood counts with automated differential profiles (15, 47). Due to the varied cellular sources of IL-6, its production was normalized to the total leukocyte count (26).

### 5.3 Assessment of mHLA-DR expression

A proteomic stabilizer (cat# 501351689, Smart Tube Inc, Las Vegas, NV) was added to collected blood prior to freezing at -80°C. At the time of mHLA-DR quantification, blood was thawed per manufacturer’s instructions. Quantibrite Anti-HLA-DR/Anti-Monocyte antibody (cat# 340827 BD Biosciences, San Diego, CA) was used to stain samples for flow cytometric quantification of mHLA-DR expression. The gating strategy for CD14+ monocytes is illustrated in **Supplementary Figure S2**. Analysis was performed using a FACS Symphony A3 (Becton Dickson & Company, Franklin Lakes, NJ) and using Flowjo v10.8.1 (BD Biosciences).

### 5.4 Statistical analysis

Using SAS (v9.4; SAS Institute, Cary, NC), with a significance threshold of 0.05, we summarized variables descriptively. The distribution of continuous variables was evaluated via histograms, probability plots, and normality tests. Demographic comparisons between groups utilized Chi-square and two-sample t-tests. We employed correlation specific to repeated measures taken over time to examine the relationships among various markers of immunoparalysis.

We used a linear mixed-effects model to compare cytokine levels between septic patients and CINS controls. Due to the non-normal distribution of cytokine data, log transformation was applied before modeling. Mean cytokine concentrations were then backtransformed to their original scale, and percentage change was calculated by exponentiating the slope estimates from the model, facilitating the interpretation of cytokine dynamics. For binary outcome variables including in-hospital mortality, 30-day mortality, 30-day readmission, and secondary infections, we used a binomial logistic regression model that included factors for each cytokine on day 1 and study group. Odds ratios overall and within each study group were used to quantify the magnitude of the relationship between each biomarker and outcome variable.

## Data Availability

The raw data supporting the conclusions of this article will be made available by the authors, without undue reservation.
