# Supplementary material for: Time-dependent variation in immunoparalysis biomarkers among patients with sepsis and critical illness
Source: Front Immunol. 2024 Dec 6;15:1498974. doi: 10.3389/fimmu.2024.1498974 (PMC11659229; doi:10.3389/fimmu.2024.1498974)
Supplement: Supplementary file 1 [file DataSheet1.pdf]

Supplementary Material

Table S1: **Microbial Sources of Sepsis**

**Septic patient**	**Primary Infectious Organism (source)**	**Clinical diagnosis of sepsis only**	**Secondary Infectious Organism (source)**
1	*Escherichia coli* (skin/soft tissue)		
2	*Staphylococcus aureus* (blood and sputum/respiratory)		
3	*Enterococcus faecalis* (urine), *Staphylococcus epidermidis* (blood)		
4	*Escherichia coli* (blood), *Klebsiella pneumoniae* (urine)		
5	*Pseudomonas aeruginosa*, *Enterococcus faecium* (urine)		
6	*Klebsiella pneumoniae* (blood)		
7	Beta *Streptococcus* group A (blood)		
8	Coagulase-negative *Staphylococcus* (blood)		SARS-COV2 (sputum/respiratory)
9	*Bacteroides thetaiotaomicron* (blood)		
10	*Haemophilus influenzae*, rhinovirus/enterovirus (sputum/respiratory)		Adenovirus (sputum/respiratory)
11	*Influenza A*, *Haemophilus influenzae*, *Staphylococcus aureus* (sputum/respiratory), *Staphylococcus epidermidis* (blood)		

**Septic patient**	**Primary Infectious Organism (source)**	**Clinical diagnosis of sepsis only**	**Secondary Infectious Organism (source)**
12	Beta *Streptococcus* group A (blood)		
13	Beta *Streptococcus* group A (blood and skin/tissue), *Staphylococcus aureus* (tissue)		
14	*Staphylococcus aureus* (blood and skin/soft tissue)		
15	*Corynebacterium striatum* (blood)		
16	*Candida glabrata*, *Rhodotorula* sp (blood)		
17		X	
18	*Proteus mirabilis* (blood and urine)		
19	*Candida glabrata* (blood and urine)		
20	*Escherichia coli* (sputum/respiratory)		
21	*Staphylococcus aureus* (blood and skin/soft tissue)		*Paracoccus yeei* (blood), *Enterococcus faecium* (skin/soft tissue)
22	*Proteus mirabilis* (blood and urine)		
23	*Enterobacter cloacae*, *Enterococcus faecalis* (abdominal)		*Pseudomonas aeruginosa*, *Enterococcus faecium*, *Providencia rettgeri* (skin/soft tissue)
24	*Escherichia coli* (blood and abdominal)		
25	*Morganella morganii* (skin/soft tissue)		

Septic patient	Primary Infectious Organism (source)	Clinical diagnosis of sepsis only	Secondary Infectious Organism (source)
26	*Klebsiella oxytoca* (blood and urine)		
27	*Klebsiella pneumoniae* (blood and sputum/respiratory)		
28	*Klebsiella oxytoca*, *Enterococcus faecalis*, *Pseudomonas aeruginosa* (urine)		
29	*Staphylococcus aureus* (blood and urine)		
30	Respiratory syncytial virus (sputum/respiratory)		
31	Respiratory syncytial virus, SARS-COV2 (sputum/respiratory), *Candida*, *Lactobacillus* species (blood)		*Enterococcus faecium* (urine)

Table S2: **Disease Etiologies of Patients with Non-Septic Critical Illness (CINS)**

CINS Patient	Primary Admission Diagnosis	Primary Discharge Diagnosis
1	Cardiogenic Shock	Acute on chronic heart failure with reduced ejection fraction
2	Critical right lower extremity limb ischemia	Same
3	Type 1 dissection of ascending aorta	Same
4	Coronary artery disease	Same
5	Seizure like activity	Altered mental status, Acute respiratory failure

**CINS Patient**	**Primary Admission Diagnosis**	**Primary Discharge Diagnosis**
6	Hyponatremia and acute encephalopathy	Same
7	Heart failure with reduced ejection fraction	Hypovolemic shock
8	Hereditary angioedema	Same
9	Acute pericardial effusion	Atrial fibrillation/flutter, Pericardial effusion
10	Pulmonary embolism with acute hypoxemic respiratory failure	Same
11	Heart failure with reduced ejection fraction status post HeartMate-3	Severe acute on chronic heart failure with LVAD with modification of LVAD parameters
12	Acute hypoxemic respiratory failure	Same
13	Acute hypoxic respiratory failure	Same
14	Trauma secondary to motor vehicle accident	Same
15	Nonischemic cardiomyopathy with cardiogenic shock	Status post heart transplantation
16	Trauma secondary to motor vehicle accident	Same
17	Traumatic brain injury	Same
18	Acute hypoxemic respiratory failure	Jejunal fistula
19	Traumatic motorcycle accident	Sinus bradycardia
20	Convulsive status epilepticus	Same
21	Subarachnoid hemorrhage	Same
22	Acute ST elevation myocardial infarction	Same
23	Acute ST elevation myocardial infarction	Same

CINS Patient	Primary Admission Diagnosis	Primary Discharge Diagnosis
24	Hilar mass with pleural effusion	Acute hypoxemic respiratory failure
25	Traumatic injury	Same
26	Acute renal failure	Acute hepatic encephalopathy
27	Trauma secondary to motor vehicle accident	Same
28	Acute epiglottis	Same
29	Hemorrhagic shock	Same
30	Fall, acute hypoxic respiratory failure	Same
31	Cardiac arrest	Same
32	Upper GI bleed	Same
33	Acute hypoxic respiratory failure, seizure disorder	Same

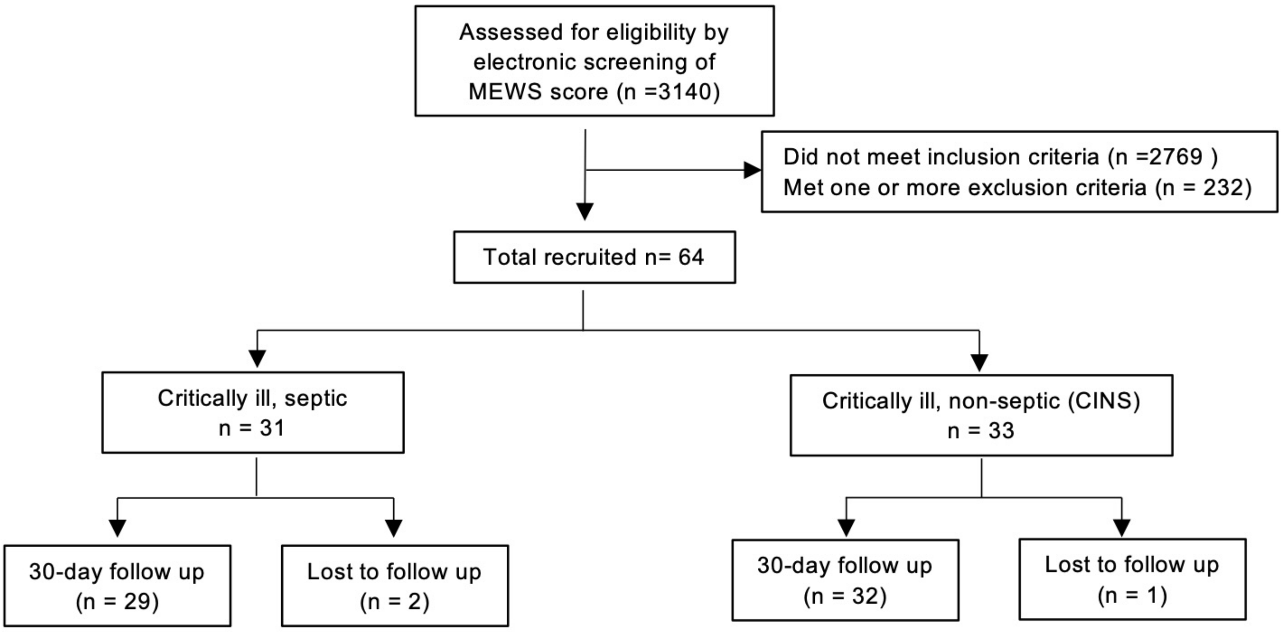

Figure S1: Flow diagram demonstrating the number of individuals included at each stage of the study.

1. Quantibrite beads are standardized fluorescent beads with known PE (phycoerythrin) molecule quantities (low, medium, medium-high, and high) that enable precise quantification of PE-conjugated antigens by converting fluorescence intensity into molecule numbers.

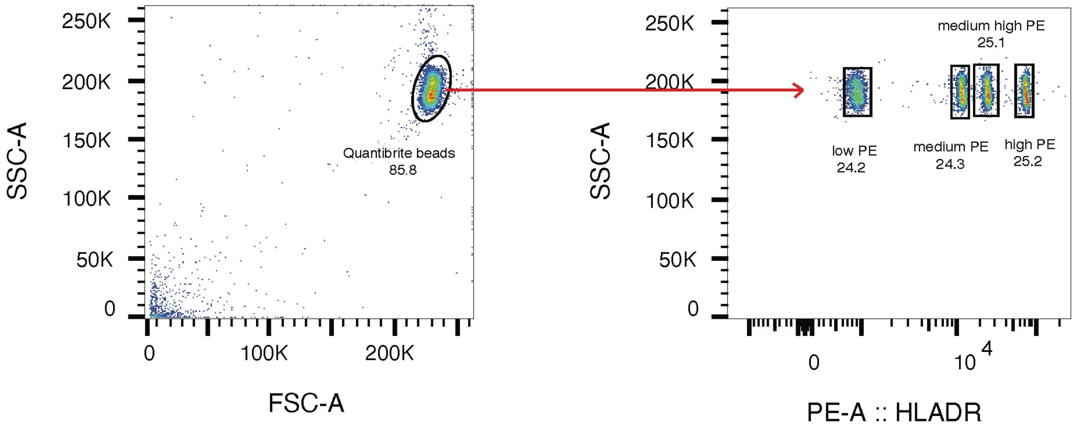

2. By measuring PE fluorescence (HLA-DR antigens) in the CD14+ subset and using a standard curve, the number of HLA-DR molecules per CD14+ cell can then be quantified.

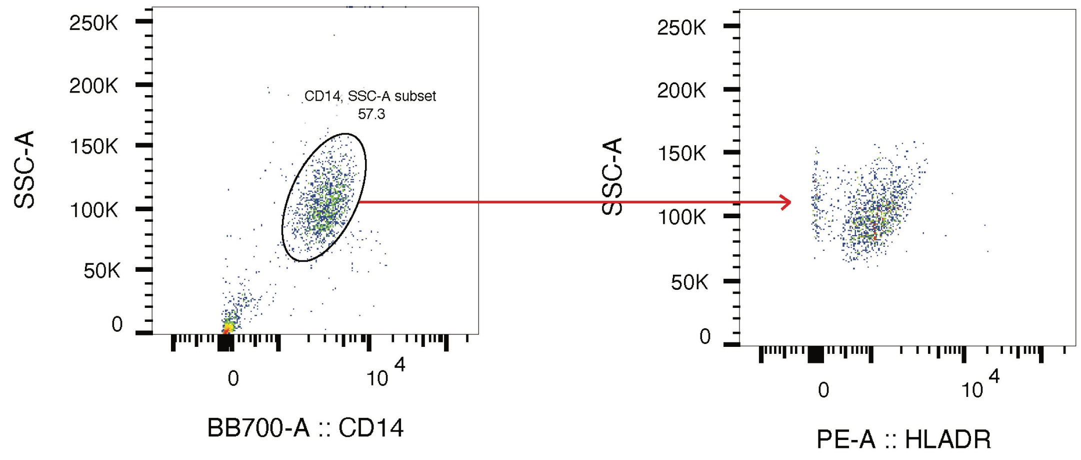

Figure S2: Flow gating to quantify monocyte HLA-DR expression using Quantibrite^{TM}.
